# The Scribble Complex PDZ Proteins in Immune Cell Polarities

**DOI:** 10.1155/2020/5649790

**Published:** 2020-04-30

**Authors:** Dante Barreda, Luis H. Gutiérrez-González, Erasmo Martínez-Cordero, Carlos Cabello-Gutiérrez, Rommel Chacón-Salinas, Teresa Santos-Mendoza

**Affiliations:** ^1^Laboratory of Autoimmunity, Instituto Nacional de Enfermedades Respiratorias Ismael Cosío Villegas, Mexico City, Mexico; ^2^Departamento de Inmunología, Escuela Nacional de Ciencias Biológicas Instituto Politécnico Nacional (ENCB-IPN), Mexico City, Mexico; ^3^Department of Virology, Instituto Nacional de Enfermedades Respiratorias Ismael Cosío Villegas, Mexico City, Mexico

## Abstract

hScrib and hDlg belong to the PDZ family of proteins. Since the identification of these highly phylogenetically conserved scaffolds, an increasing amount of experiments has elucidated the roles of hScrib and hDlg in a variety of cell functions. Remarkably, their participation during the establishment of polarity in epithelial cells is well documented. Although the role of both proteins in the immune system is scantly known, it has become a growing field of investigation. Here, we summarize the interactions and functions of hScrib and hDlg1, which participate in diverse functions involving cell polarization in immune cells, and discuss their relevance in the immune cell biology. The fundamental role of hScrib and hDlg1 during the establishment of the immunological synapse, hence T cell activation, and the recently described role of hScrib in reactive oxygen species production in macrophages and of hDlg1 in cytokine production by dendritic cells highlight the importance of both proteins in immune cell biology. The expression of these proteins in other leukocytes can be anticipated and needs to be confirmed. Due to their multiple interaction domains, there is a wide range of possible interactions of hScrib and hDlg1 that remains to be explored in the immune system.

## 1. Introduction

Proteins play a fundamental role in cellular functions, associating themselves with lipids, nucleic acids, and other proteins. The capability of protein-protein interaction is determined by the presence of structural subunits (domains) able to work in a relatively independent way from the rest of the protein in a stable or transitory manner, regulating a wide range of cellular functions [1, 2].

PDZ (postsynaptic density (PSD95), discs large (Dlg), and Zonula occludens (Z0-1)) domains are very common in protein-protein interactions in a broad range of eukaryotic organisms [3]. The importance of proteins containing PDZ domains (PDZ proteins) for cellular homeostasis could be anticipated since they are highly conserved between species and are involved in a vast diversity of cellular functions [4]. In humans, several PDZ proteins have been identified and their functions are well documented, mainly in neuronal and epithelial cells.

The Scribble complex contains two PDZ proteins: Scrib and Dlg. The Lgl (Lethal-2-giant larvae) protein is the third member of the Scribble complex but does not possess PDZ domains. The Scribble complex has been widely studied for its function in the establishment of apicobasal epithelial cell polarity (ABCP) [5].

Several years ago, the expression and participation of the Scribble complex in the establishment of the uropod and immunological synapse were described in T lymphocytes [6]. More recently, the fundamental function of hScrib for reactive oxygen species (ROS) production in macrophages (M*ϕ*) and of hDlg1 for cytokine secretion by dendritic cells (DCs) has been described [7, 8]. Moreover, we have recently reported the expression and regulation of hScrib and hDlg1 in human APC (antigen-presenting cells) including DCs, M*ϕ*, and monocytes [9], supporting that both proteins are involved in fundamental processes of the immune response.

In this work, we summarize the functions of the Scribble complex with particular attention to hScrib and hDlg focusing on cells of the immune system and discuss the potential role of these proteins during the immune response.

## 2. PDZ Proteins

Previously identified as GLGF (Gly-Leu-Gly-Phe) or DHR (discs-large homology region) domain, the PDZ domains were originally recognized as 80-90 amino acid repeated sequences in the MAGUK (membrane-associated guanylate kinase) proteins PSD-95, Dlg, and ZO-1, hence its name [3, 10]. PDZ domains are commonly found in tandem and associated with other interaction domains to form multidomain scaffold proteins. PDZ domain consists of six *β*-strands flanked by two *α*-helix that form a pocket where the interaction with its ligand occurs. PDZ domains usually bind to short specific peptide sequences located at the C-terminal of their partner proteins known as PDZ binding motifs (PDZbm). PDZbms have been classified into three groups: the type I PDZbm has the consensus sequence X-S/T-X-V/I/L_COOH_, type II has the consensus sequence X-*ϕ*-X-*ϕ*, and type III has the consensus sequence X-D-X-V (X: any amino acid; *ϕ*: a hydrophobic amino acid). In addition, PDZ domains can also interact with internal peptide motifs and certain lipids and can take part in a head-to-tail oligomerization with other PDZ domains [3, 4].

PDZ proteins are highly conserved through living organisms ranging from bacteria to mammals. The PDZ-containing genes encoded in archaea, plants, and fungi are vastly outnumbered by those in bacterial and metazoan genomes. To date, more than 333,000 PDZ domains in 225,000 proteins are registered in the SMART nonredundant database (http://smart.embl-heidelberg.de/), with 98,693 proteins in metazoans and 117,402 in bacteria. The human genome contains 152 PDZ protein-coding genes reported so far, many of them expressing several isoforms (HUGO Gene Nomenclature Committee at the European Bioinformatics Institute; https://www.genenames.org/data/genegroup/#!/group/1220).

According to their modular organization, PDZ proteins can be classified into three families: (1) proteins consisting exclusively of PDZ domains ranging from one to more than 10; (2) the MAGUK family characterized by containing one or three PDZ domains, an SH3 domain, and a GUK-like domain (without catalytic activity); (3) proteins that contain PDZ domains and other protein domains such as ankyrin, LIM (Lin-1, Isl-1, and Mec-3), WW (WWP repeating), and LRR (leucine-rich repeat) among others [4].

Generally, PDZ proteins contain a large number of noncatalytic domains that allow them to interplay through protein-protein interactions; hence, their main function is to act as scaffolds able to hold receptors and signaling molecules assembling supramolecular complexes. Depending on the proteins in the complex, as well as on its subcellular localization, PDZ proteins organize a vast range of cellular functions. One of the most studied complexes that include PDZ proteins is Scribble, which plays major roles in cellular polarity.

## 3. The Scribble Complex

### 3.1. The Scribble Complex in Drosophila

Scrib, Dlg, and Lgl are the three components of the Scribble complex. These proteins were first identified in *Drosophila melanogaster* as tumor suppressors, and later, their participation in cellular polarity was discovered [11].

The gene *scrib* was first identified in genetic screening of maternal mutations that resulted in altered larva epithelial morphogenesis in *Drosophila*. These mutants produce embryos with a corrugated cuticle, instead of the smooth cuticle of wild-type embryos, therefore the name scribble [11]. Later, the correlation between *scrib*, *dlg*, and *lgl* genes was established, whose individual mutations produced a similar phenotype of the *scrib* mutants. Mutations in these genes resulted in tissue-specific tumors (discs large (*dlg*)) or the complete larva overgrowth, causing death (lethal giant larvae (*lgl*)). These genes function as a complex; Dlg or Lgl mutants intensified the Scrib phenotype, which points to a single genetic pathway [12].

Scrib, Dlg, and Lgl do not possess intrinsic enzymatic activity; these proteins are characterized by the presence of several protein-protein interaction domains; thus, they function mainly as scaffolds to assemble multiprotein complexes that take part in diverse cellular functions such as protein trafficking, signaling, and cellular polarization [4].

*scrib* encodes Scrib, a 195 kDa protein classified into the LAP (LRR and PDZ domain) family of proteins that contains 16 LRR motifs, two LAPSD (LAP-specific) domains, and four PDZ domains [12]. Dlg is a 102 kDa protein, whose amino acid sequence includes an arrangement of several domains conserved among the MAGUK protein family: three PDZ domains, an SH3 domain, and a GUK-like domain [13]. Lgl is a 130 kDa protein that contains several WD40 (tryptophan-aspartic acid dipeptide) motifs often involved in the coordination of supramolecular protein complex assembly [14]. Proteins from the Scribble complex are highly conserved throughout species, both in sequence and function. For instance, the mammalian Dlg is capable of rescuing *Drosophila*'s mutant phenotype [15].

### 3.2. The Human Scribble Complex

**Figure 1:**
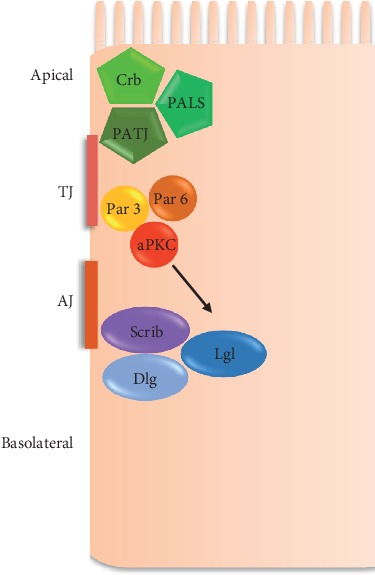
ABCP polarity complexes in mammalian epithelial cells. Protein complexes of the apicobasal epithelial cell polarity are illustrated. Crumbs is concentrated in the apical region, Par3 in the tight junction (TJ), and Scribble in the adherens junction (AJ) and basolateral region. hLgl localization is controlled by aPKC phosphorylation.

Only one gene for *scrib* (hScrib/SCRIB) has been found in humans, while for *dlg* and *lgl*, five and two genes have been reported, respectively (Table 1). The Scribble complex localizes to the basolateral membrane in epithelial cells; each of the proteins is required for proper localization of the other PDZ protein complexes involved in apicobasal polarity: Crumbs and Par [5, 16–18] (Figure 1).

#### 3.2.1. hScrib

**Figure 2:**
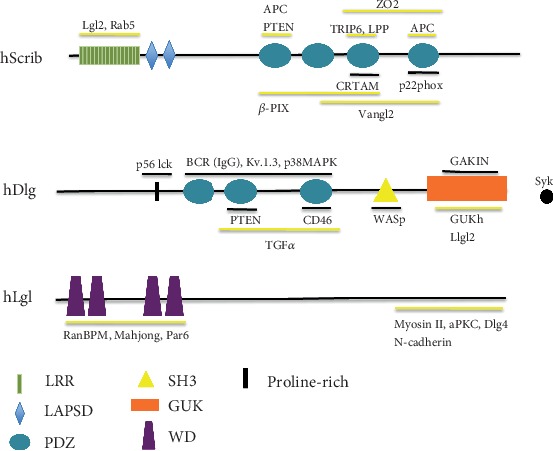
The members of the human Scribble complex. The domain composition of each protein is illustrated. Described interactions with specific domains are indicated. Black lines: interactions described in immune cells; yellow lines: interactions described in other cell types; black dot: Syk interaction with Dlg1 through unknown domain.

The human Scrib was identified in a biochemical screening looking for protein targets of the E6 oncoprotein of the human papillomavirus 16 (HPV 16). *scrib* can express two isoforms (Table 1). Similar to the *Drosophila* protein, hScrib (220 kDa) has 16 LRR motifs and four PDZ domains [19] (Figure 2). LRRs determine its ability to localize to the basolateral epithelial membranes, and the PDZ domains allow its interaction with different proteins expressing PDZbms (Table 2). For example, the third and fourth PDZ domains of hScrib interact with a PDZbm encoded by ZO2; such interaction is necessary for ZO2 proper functioning at the tight junction of epithelial cells [20].

The expression of *scrib* is low in the kidneys, skeletal muscle, liver, and lungs and high in the breasts, bowels, placenta, and skin. The highest expression levels of hScrib have been observed in epithelial cells (https://www.ncbi.nlm.nih.gov; GeneID: 23513).

#### 3.2.2. hDlg

The five *dlg* genes reported in the human genome can express 23 isoforms (Table 1) that encode proteins with molecular weights ranging from 80 to 200 kDa. All the hDlg isoforms share the basic structure of the MAGUK protein family. Additionally, specific domains can be found that distinguish the different isoforms. For instance, some isoforms of hDlg1 possess a L27 (Lin-2 and Lin-7 proteins) domain in its amino-terminal (N-terminal) that is not present in the other hDlg proteins (https://www.ncbi.nlm.nih.gov; GeneID:1739). Human Dlg1 is homologous to *Drosophila* Dlg and is mainly expressed in epithelial cells; as it has been extensively studied, we will focus henceforth on hDlg1 [21].

hDlg1 domains are responsible for a large number of specific interactions of the protein. For example, the L27 (Lin-2 and Lin-7 proteins) domain makes hDlg1 capable of interacting with other MAGUKs via homodimeric interactions. On the other hand, the SH3 domain binds in an intramolecular fashion to the GUK domains, preventing interactions with other MAGUKs [22, 23]. Regarding PDZ domains, they allow hDlg1 to associate with PDZbm-bearing proteins. For example, the second PDZ domain of hDlg1 binds the PDZbm of PTEN (phosphatase and tensin); this interaction inhibits the PI3K (phosphatidyl inositol 3 kinase) pathway and cytoskeleton reorganization of epithelial cells [24] (Figure 2).

*dlg1* transcripts have been found in the brain, skeletal muscle, kidney, liver, cardiac muscle, and lungs. In addition, it is highly expressed in the basement membrane of bowel cells (https://www.ncbi.nlm.nih.gov; GeneID: 1739).

#### 3.2.3. hLgl

The genes *lgl1* and *lgl2* encode a 115 kDa and a 113 kDa proteins, with one and three isoforms, respectively (https://www.ncbi.nlm.nih.gov; GeneID:3996 and 3993, respectively). As in *Drosophila*, the N-terminal sequence of the human Lgl contains several WD40 motifs that form *β* strands that act as interaction protein modules. Indeed, WD40 motifs function as a scaffold in numerous cellular functions like migration, polarity, and cell adhesion [25].

While the *lgl1* gene is expressed in the brain, testis, ovaries, and endometrium, *lgl2* is present in the colon, stomach, and small intestine (https://www.ncbi.nlm.nih.gov; GeneID: 3996 and 3993, respectively). In mammalian epithelium, both hLgl1 and hLgl2 are localized at the lateral membrane underlying tight junctions. This localization depends on its phosphorylation by an aPKC (atypical protein kinase C) during the establishment of epithelial polarity [26].

The carboxyl-terminal (C-terminal) region of hLgl participates directly in protein-protein interactions, for example, with N-cadherin, which is fundamental to the formation of apical junctional complexes necessary for cell adhesion in embryonic stem cells [27].

## 4. The Scribble Complex in Polarity Processes

### 4.1. Polarity in Epithelial Cells

The membrane and cytoplasmic content of every single cell must be asymmetrically distributed to perform routine functions such as transport and absorption of molecules, interaction with the environment, and cell migration. This molecular segregation is known as cellular polarity [28, 29].

There are four main types of cellular polarity: (1) apicobasal cell polarity (ABCP, epithelial polarity), (2) planar cell polarity (PCP, polarity in the epithelium sheet plane), (3) front-rear polarity (FRCP, involved in cell migration), and (4) asymmetric cell division (involved in self-renewal of stem cells) [30]. Additionally, cells with high specialized functions show variants of these four types of polarity. Several protein complexes participate in the establishment and maintenance of cellular polarity. Scribble, PAR3, and Crumbs complexes dictate ABCP through the recruitment of surface receptors and signaling molecules to the membrane, the interaction with the cytoskeleton, and other structural elements of the cell and by affecting the location and activity of other polarity complexes [5]; these three complexes contain PDZ proteins.

Experiments carried out in *Drosophila* and mammals have proven that the localization of each polarity complex is influenced by the other complexes. An example is the regulation between Scribble and PAR3 complexes. In epithelial cells, hLgl interacts with PAR6 at cell-cell contact sites and, after phosphorylation by PKC*ζ* (protein kinase C zeta), hLgl dissociates from PAR3 complex moving towards basolateral membranes to establish ABCP [26] (Figure 1).

The concept of “cellular fitness,” derived from the theory of cell competition in developmental biology, is defined as the ability of a cell to prosper in a given environment; it is determined by several parameters, such as signaling activity and cell-cycle length [31]. In addition to classical polarity functions such as migration and barrier formation, the ABCP polarity protein complexes take part in transient molecular segregation to carry out functions such as apoptosis, cell signaling, or proliferation elicited by environmental stimuli. “Cellular fitness functions” have been proposed to refer to the functions in which molecular subcellular polarization, organized by polarity protein complexes, occurs [29].

### 4.2. Polarity in Immune Cells

Mechanisms regulating epithelial cell polarity have been widely studied. It is thought that, despite the biological and functional differences between epithelial and immune cells, fundamental principles that determine polarity are highly conserved [32]. Homeostatic leukocyte migration throughout the body or to specific sites of infection implies the directed movement toward gradient cues, for which many receptors and adhesion molecules should be polarized in a specific manner [33]. Scribble complex, one of the three ABCP-controlling complexes, plays fundamental roles in several lymphocyte functions such as T cell migration, proliferation, homotypic interactions, and antigen-induced activation [6, 34, 35].

## 5. The Scribble Complex in Lymphocyte Functions

T and B lymphocytes are the distinctive cells of the acquired immune response. Adaptive immunity starts with the activation of T cells that recognize the antigen (Ag) in a very precise manner through clonally distributed receptors. To achieve this, T cells perform a rearrangement of the membrane and subcellular components such as surface receptors, signaling complexes, and cell organelles. Two of these events are the uropod formation and the immune synapse establishment [36].

### 5.1. Role of the Scribble Complex in T Cell Migration

During migration, T cells display a characteristic morphology, with the formation of the leading edge and the uropod, to which specific molecules are segregated. Polarity dictates not only the cell directional movement but also cytoskeleton coordinated modifications necessary for propulsion. Migrating T cells have a protruding structure at the rear, the uropod, which contains the microtubule-organizing center (MTOC) and is rich in adhesion molecules [37].

hScrib and hDlg1 polarize to the uropod during T cell migration. Downregulation of hScrib makes T cells unable to form the uropod, and consequently, these cells show inefficient migration. In addition, these cells are unable to polarize hDlg1, Ezrin, and CD44 proteins that normally polarize towards the uropod [6]. Altogether, these findings support the role of hScrib and hDlg1 during T cell uropod formation and in the orchestration of the polarization of other T cell proteins.

### 5.2. The Scribble Complex in the Immune Synapse and T Cell Activation

The immunological synapse (IS) is the structure formed in the interface between APCs and T cells, where T cell receptors (TCR) recognize their cognate Ag as MHC- (major histocompatibility complex-) peptide complex on the surface of an APC. The word “synapse” was taken from its homology with the neuronal synapses. Both structures, immune and neuronal synapses, are formed with high molecular specificity to transfer information [38]. It has been known for years that the organization of excitatory neuronal synapses requires the scaffolding functions of PDZ proteins. For instance, PSD-95 (Dlg4) is highly expressed and is the best-characterized PDZ protein on the postsynaptic density (PSD) [39]. Indeed, Dlg4 is necessary for the organization of NMDA (N-methyl-D-aspartate) receptors; hence, the glutamatergic signaling transduction pathway [40].

The adaptive immune response initiates with the activation of naïve T cells by DCs through Ag presentation. Usually, Ag presentation occurs in the regional lymph node. T cells and DCs migrate to the lymph node paracortex, where T cells screen multiple DCs to find its cognate Ag and to establish an IS. In this cellular encounter, polarized processes play a crucial role for a proper IS formation, in which molecular segregation should take place at both sides of the synapse. Several proteins are recruited, and others excluded from the IS with great precision. For instance, the MTOC is recruited towards the IS, while other molecules such as Ezrin and CD43 are excluded to the distal pole [6, 41, 42]. This molecule segregation is based on an appropriate polymerization of F-actin in both DCs and T cells, which is fundamental for the IS formation and T cell activation [6].

Early during the IS formation, both hDlg1 and hScrib relocalize to the IS in T cells, to later move away towards the distal pole. During these processes, hScrib and hDlg1 colocalize, consistently with the cooperative role that is attributed to these proteins. In addition, hScrib is necessary for the IS establishment. hScrib downregulation in CD4^+^ T cells induces fewer T-DC conjugates than wild type. Moreover, these cells are unable to polarize CD3 and PKC*θ* after stimulation via CD3/CD28, resulting in the inhibition of TCR response [6].

After activation, T cells proliferate to produce effector progeny, which migrate to the activation site. These effector cells perform functions such as the direct killing of their target cells (cytotoxicity) and the coordination of immune response through direct cell-to-cell contact and cytokine secretion [43].

**Figure 3:**
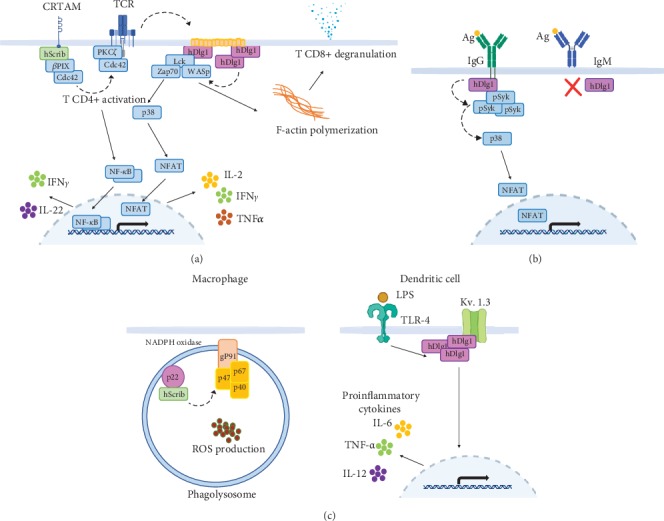
The Scribble complex in immune cells. (a) Some functions of hScrib and hDlg1 during T cell activation are illustrated. TCR stimulation induces the interaction of hScrib with CRTAM; in turn, Cdc42 and PKC*ζ* are recruited to the TCR to regulate IFN*γ* and IL-22 secretion. hDlg1 is constitutively found in lipid rafts (orange) associated with Lck, Zap70, and WASp. After TCR stimulation, additional hDlg1 molecules are recruited, activating the p38 MAPK/NFAT pathway to induce cytokine production. Additionally, F-actin polymerization mediated by WASp within the same complex is involved in T cell degranulation. (b) hDlg1 regulates B cell memory responses. The PDZ-dependent interaction of hDlg1 with the BCR IgG is increased upon Ag recognition leading to pSyk recruitment and p38 MAPK activation stabilizing IgG signaling. (c) hScrib and hDlg1 regulate APC functions. In M*ϕ* (left), hScrib is necessary for the assembly of the NADPH oxidase complex and ROS production. In DCs (right), hDlg1 participates in cytokine production upon TLR stimulation through the stabilization of potassium channel expression on the plasma membrane. Solid arrows represent signaling events while dashed arrows represent recruitment events (see text for details).

In T cells, hDlg1 is constitutively found in lipid rafts associated with WASp (Wiskott-Aldrich syndrome protein), Lck (lymphocyte-specific protein tyrosine kinase), and Zap70 (Zeta-chain-associated protein kinase 70). hDlg1 location to plasma membrane appears to be dependent on its interaction with the SH3 domain of Lck. hDlg1 augments the association of this complex with activated Zap70 in response to TCR activation. The formation of this complex appears to rely on hDlg1 scaffold activity since Lck-WASp and Lck-Zap70 complexes are reduced in *dlg1*-downregulated cells. The F-actin polymerization in T cells after activation is mediated by WASp and depends on the hDlg1/Lck/Zap70 complex formation [44] (Figure 3).

In addition, two hDlg1 isoforms differentially regulate CD8^+^ T lymphocyte effector functions. hDlg1AB, by its interaction with Lck, promotes p38 MAPK (P38 mitogen-activated protein kinase) activation, resulting in NFAT translocation to the nucleus thus IFN*γ* and TNF*α* production. On the other hand, both hDlg1AB and hDlg1B are required for cytotoxic lymphocyte degranulation independently of p38 MAPK activation but depending on hDlg1-WASp complex formation [45]. Furthermore, Ag stimulation of hDlg1-downregulated CD8^+^ T cells results in reduced IFN*γ* and IL-2 production [44]. Taken together, these data point to a positive regulatory role for hDlg1 in T cell activation. Additionally, Xavier et al. observed the recruitment of hDlg1 to the IS but, in apparent contradiction with Round et al.'s work [44], they found hDlg1 to be a negative regulator of T cell NF-AT (nuclear factor of activated T cells) activation and proliferation [46]. These apparent discrepancies might be explained by the use of different experimental approaches.

In a late phase after TCR stimulation (14 hours), the CD4^+^ or CD8^+^ CD62L^+^ T cell subsets upregulate the transmembrane receptor class-I MHC-restricted T cell-associated molecule (CRTAM) [34]. In its cytoplasmic tail, CRTAM contains a PDZbm that interacts with the third PDZ domain of hScrib. This interaction induces the recruitment of Cdc42 and PKC*ζ* towards TCR and is indispensable for the assembly of a multiprotein complex that biases the T cell response toward the production of the proinflammatory cytokines IFN*γ* and IL-22 instead of proliferation [34] (Figure 3). Moreover, hScrib interacts indirectly with Cdc42 by means of *β*PIX (*β*PAK-interacting exchange protein) which controls the activation and localization of this GTPase (guanosine triphosphatase) [47]. When hScrib/CRTAM complexes are absent, Cdc42 polarization fails. Hence, hScrib-/- Th1 lymphocyte response after primary TCR stimulation is deficient [34].

Most of the above-described functions of hScrib and hDlg1 in immune cells have been elucidated using mainly siRNAs and shRNAs. hScrib and hDlg1 knockout (KO) mice are not viable. Therefore, different experimental approaches, such as chimeric or conditional KO mice, have been used to study their deficiency *in vivo*. Most of these studies have been focused on hDlg1 in T cells, and all of them conclude that hDlg1 is dispensable during T cell development, while mature T cells display slight or no phenotypes during stimulation [48, 49]. An explanation could lie in a compensatory mechanism involving the other hDlg genes. hDlg1-/- mature T cells obtained from KO mice elicit attenuated responses after TCR stimulation compared with those obtained using siRNAs [48–50]. Notably, in a conditional hDlg1 KO model, TCR stimulation of mature T cells induces hyperproliferation [48], reinforcing the idea of hDlg1 as a negative regulator of T cell proliferation. It has been hypothesized that there is a short window during development in which the selection of cells that have compensated for hDlg1 ablation occurs (likely by additional hDlg isoforms), thus giving rise to mature T cells with attenuated responses in germ-line KO mice. This suggests that there is a checkpoint during development, in which compensation for hDlg1 absence would occur [48, 49]. In light of these analyses, the use of siRNAs and molecules that block the function of these proteins has shown to be the best approach to study the lack of hScrib and hDlg1 until more suitable conditional KO models are developed. Their use has shown differences in phenotypes and functions in lymphocytes, M*ϕ*, and DCs, which makes them a good tool to explore the functions of both proteins in immune cells [6, 7, 44, 45, 51]. With an alternative experimental approach, it has been suggested that Scrib deficiency partially blocks an early stage of double-negative (DN) thymocyte differentiation, affecting T cell development, with a delayed transition from the early to late DN3 stage [35]. Additionally, the cell-to-cell contacts necessary for adequate T cell development are impaired due to a failure in appropriate LFA (lymphocyte function-associated) polarization that affects homotypic interactions and clustering. In this approach, Scrib was knocked down in fetal liver hematopoietic precursors that were then subjected to an *in vitro* T cell differentiation model [35].

### 5.3. The Scribble Complex in T Cell Memory Responses

Memory is the hallmark of adaptive immunity. After the effector phase of an immune response, most T cells die leaving a small proportion of memory T cells that remain for many years and are capable to respond quickly to another challenge with the same Ag [52]. Two subpopulations of memory cells have been described: effector memory T cells (T_EM_) that have high migration potential and display immediately effector functions and central memory T cells (T_CM_), which locate in T cell zones of secondary lymphoid tissues and do not have effector functions but quickly proliferate and differentiate into effector memory T cells in response to Ag [53].

Using a conditional KO model, with deletion of Dlg1 restricted to T lymphocytes, it was demonstrated that Dlg1 is required for the normal generation of memory T cell subsets *in vivo* [50]. Dlg1-deficient mice show a higher percentage of T_CM_ CD4^+^ T cells and a lower percentage of T_EM_ CD4^+^ T cells compared to wild-type mice. Even though the mechanism by which this phenotype is achieved remains unknown, it has been suggested that potassium channels might be involved [50]. Dlg1 interacts in a PDZ dependent-manner with Kv.1.3 subunit regulating the function of potassium channels at the plasma membrane. On the other hand, it has been reported that loss of function of potassium channels results in the reversion from T_EM_ into T_CM_ phenotype [54, 55]. Additionally, *in vitro* recall responses show an increased number of IL-2-producing T cells in Dlg1 KO mice compared to WT. Altogether, these results suggest that hDlg1 is involved in the regulation of T cell memory responses.

### 5.4. The Scribble Complex in B Cells

The humoral immune response is initiated when naïve B cells recognize their cognate Ag through the B cell receptor (BCR) complex expressed on the cell surface, where clonotypic IgM functions as an Ag receptor. After the first encounter with its specific Ag, the B lymphocyte becomes fully activated after establishing cooperation with a CD4^+^ T cell. This leads to B cell proliferation and generation of both memory B cells and antibody-producing plasmatic cells, which have different Ig subclasses with a higher affinity for the Ag. Mature effector B cells switch their receptor from IgM into IgG or other isotypes, depending on the cytokine environment induced by CD4^+^ T cells. Memory B cells retain the IgG receptor and respond to a reencounter with the same Ag differentiating rapidly into plasmatic cells producing abundant antibodies [56].

Interestingly, the Ig*α*/*β* chains of the IgG-BCR contain signaling motifs in their cytoplasmic tail that are absent in IgM-BCR [57]; after the initial interaction of the IgG-BCR with its cognate Ag, hDlg1 is recruited to the IS through a PDZ-dependent interaction with the IgG-BCRs that contain a PDZbm in their cytoplasmic tail. After IgG-BCR engagement, the “capping” of BCRs along with phosphorylated spleen tyrosine kinase (pSyk), one of the first kinases recruited to the IS, appears to be dependent on hDlg1, since a lower amount of pSyk and BCRs is recruited in hDlg1 knockdown B cells [57]. In addition, hDlg1 is involved in p38 MAPK activation in B cells; thus, hDlg1 downregulation results in lack of p38 MAPK phosphorylation and weaker retention of IgG-BCRs on the membrane, resulting in lower cell activation [57]. In contrast, hDlg1 does not participate in primary B cell responses, because IgM does not induce hDlg1 recruitment after stimulation, due to the absence of a cytoplasmic tail and therefore a PDZbm (Figure 3). Accordingly, Dlg1 deficiency in B cells from chimeric mice did not affect proliferation and isotype switching during primary responses [58]. Interestingly, secondary antibody responses in these chimeric Dlg1-deficient mice appear to be unaltered compared to those of wild-type mice [58]. It is possible that the reduction in IgG signaling due to the absence of Dlg1 does not impact antibody production *in vivo*. The possibility that different hDlg isoforms expressed in B cells might compensate for the absence of hDlg1 should be analyzed. In addition, KO chimeric mice deficient in Scrib or Dlg1 display unaltered B lymphocyte development and have no effect on *in vivo* B cell responses to immunization and challenge with influenza virus. Nevertheless, Scrib deficiency promotes a slight but significant increase in CD138^+^ plasma cells after B cell stimulation with LPS [58].

## 6. The Scribble Complex in Antigen-Presenting Cells (Myeloid Lineages)

### 6.1. Scribble Complex in Dendritic Cells

DCs are the bridge between innate and adaptive immunity. They stay immature (iDCs) until they encounter, capture, and process Ag, which lead them to a maturation process. Mature dendritic cells (mDC) are characterized by high expression of costimulatory molecules and cytokines necessary for proper naïve T cell activation [59].

In addition to Ag presentation, DCs deploy an array of different functions such as migration, phagocytosis, and cytokine and ROS production. These events require transitory cell polarization and supramolecular complex formation, which suggest the participation of PDZ proteins and can be grouped as “cellular fitness functions” [29, 60–63].

The PDZ protein Spinophilin is expressed in neuron dendrites, in synaptic junctions, and adherens junctions in the epithelium [64, 65]. Low expression levels of Spinophilin have been associated with several types of cancers, such as glioblastoma, lung carcinomas, and colon cancer malignant phenotype, and with poor prognosis in breast tumors [66, 67]. Spinophilin is also expressed in DCs; it localizes throughout iDC cytoplasm and relocalizes to the plasma membrane during DC maturation and to the IS during Ag presentation, where it is required for optimum T cell activation [68].

Recently, we reported the expression and regulation of hScrib and hDlg1 in monocytes, M*ϕ*, and DC [9]. Similar to Spinophilin, hScrib relocalizes to the plasma membrane during DC maturation.

In addition, we found that expression of both hScrib and hDlg1 increases when iDCs mature to mDCs while hScrib, but no hDlg1, is polarized towards the IS in DC during Ag presentation. Furthermore, we demonstrated the absence of hScrib and hDlg1 colocalization in iDCs and mDCs, which suggests that these proteins are not forming a complex and, possibly, they are participating in different pathways in these cells [9]. Recently, hDlg1 has been implicated in several DC functions such as Ag uptake and cytokine secretion. The PDZ-dependent interaction of hDlg1 with Kv 1.3 potassium channels appears to be relevant in the regulation of such processes [8] (Figure 3). Altogether, these experimental data suggest an important role of the Scribble complex in the functioning of DCs.

### 6.2. The Scribble Complex in Macrophages

M*ϕ* are professional APCs, found in all tissues, which display a wide range of functions. They are avid phagocytic cells with enormous killing power. The NADPH (nicotinamide adenine dinucleotide phosphate) oxidase complex in M*ϕ* comprises membrane-bound (p22phox, gp91phox) and cytosolic components (p67phox, p47phox, and p40phox). This complex is rapidly assembled after several stimuli. The active NADPH oxidase leads to the reduction of O_2_ for reactive oxygen species production that directly damages microbes [69].

It has been recently shown that Scrib is indispensable for the NADPH oxidase complex assembly in M*ϕ*. The fourth PDZ domain of Scrib specifically interacts with the p22phox subunit of NADPH complex which contains a PDZbm. This interaction is fundamental for (ROS) production, since Scrib-downregulated M*ϕ* show impaired ROS production and consequently are less efficient to kill pathogens [7] (Figure 3).

## 7. Concluding Remarks

Polarity protein complexes are part of the multiple molecule arrangements and finely regulated functions that occur within the immune cells to maintain their homeostasis and activity. The concept of cellular fitness, derived from the Darwinian principles of cell competition and adaptation, is especially useful to understand the effects that this subcellular molecular polarization and its alterations may have in the immune response. The PDZ proteins hScrib and hDlg1, for example, partake in many pivotal functions of leukocytes from both lymphoid and myeloid lineages. The formation of polarized structures, such as the uropod and immune synapse, contributes significantly to the adjustment of immune cellular fitness. In addition to affecting structural polarities in these highly motile cells, hScrib and hDlg1 may also affect functional polarities that contribute to modification of immune responses. For example, hScrib participates in the assembly of the NOX2 complex and, consequently, in ROS production. According to their role as scaffolds, hScrib and/or hDlg1 also take part in the construction of numerous complexes involved in signaling pathways with important effector functions in leukocytes, such as cytokine production and proliferation. Given that both hScrib and/or hDlg1 are highly conserved in sequence and function, it is reasonable to think that these proteins may have similar functions in other leukocytes. In the future, it will be interesting to explore whether these proteins are expressed in other leukocytes, the interactions they may establish, and the outcome of such possible interactions. Interestingly, it has been recently hypothesized that the kidnapping of PDZ proteins by viral PDZbm-bearing proteins may be a common mechanism of immune evasion [70]. These possibilities make the study of PDZ polarity proteins all the more important.

## Figures and Tables

**Table 1:** The human Scribble complex genes (source: https://www.ncbi.nlm.nih.gov/gene).

Gene	Official name	Chromosomal location	Exons	Aliases	Tissue of expression	Gene isoforms	GeneID
*Scrib*	Scribble planar cell polarity protein	8	37	CRIB1, SCRB11, Vartul, SCRIB	Colon, testis, skin, and kidney	2	23513
*Dlg1*	Discs large MAGUK scaffold protein 1	3	35	DLGH1, SAP-97, SAP97, dJ1061C18.1.1, hDlg	Thyroid, brain, and esophagus	15	1739
*Dlg2*	Discs large MAGUK scaffold protein 2	11	45	PPP1R58, PSD-93, PSD93, chapsyn-110	Brain	9	1740
*Dlg3*	Discs large MAGUK scaffold protein 3	13	26	MRX, MRX90, NEDLG, PPP1R82, SAP102, XLMR	Brain, colon, and thyroid	3	1741
*Dlg4*	Discs large MAGUK scaffold protein 4	17	27	PSD95, SAP-90, SAP90	Brain	6	1742
*Dlg5*	Discs large MAGUK scaffold protein 5	10	43	LP-DLG, P-DLG5, PDLG	Placenta, skin, adrenal glands, and thyroid	1	9231
*Llgl1*	LLGL1, scribble cell polarity complex component	17	23	DLG4, HUGL, HUGL1, LLGL, Lgl1, Mgl1	Brain, testis, ovaries, and endometrium	1	3996
*Llgl2*	LLGL2, scribble cell polarity complex component	17	31	HGL, Hugl-2, LGL2	Colon, stomach, and small intestine	3	3993

**Table 2:** hScrib and hDlg1 interacting proteins in immune cells. Interactions of hScrib and hDlg1 in specific immune cells and functional outcome of each interaction are shown.

PDZ protein	Immune cell	Interacting protein	Interacting domain	Function	Reference
Scrib					
	M*ɸ*	p22 phox	PDZ4	NADPH complex assembly, ROS production	[7]
	T cell (CD4^+^ and CD8^+^)	CRTAM	PDZ3	Cdc42/PKC*ζ* polarization, cytokine production	[34]

Dlg1					
	Treg	PTEN	PDZ	Akt and NF-*κ*B inhibition	[71]
	T CD8^+^	p56 Lck	Prolin rich	Zap 70 and p38 MAPK activation	[45]
	T CD8^+^	WASp	SH3	F-Actin polymerization, degranulation	[45]
	T cell	Zap 70	—	Scaffold	[44]
	T cell	WASp	—	Scaffold	[44]
	T cell	p38 MAPK	PDZ	NFAT activation	[72]
	T cell	p56 Lck	Prolin rich	Scaffold	[54]
	T cell	Kv 1.3	PDZ	Scaffold	[54]
	T cell	GAKIN	GUK	Intracellular trafficking	[73]
	B cell	BCR (IgG)	PDZ	p38 activation BCR-downstream signaling	[57]
	DC	Kv 1.3	PDZ	Cytokine production	[8]
